# Mariner mutagenesis of *Brucella melitensis *reveals genes with previously uncharacterized roles in virulence and survival

**DOI:** 10.1186/1471-2180-6-102

**Published:** 2006-12-18

**Authors:** Qingmin Wu, Jianwu Pei, Carol Turse, Thomas A Ficht

**Affiliations:** 1 Department of Preventive Veterinary Medicine, College of Veterinary Medicine, China Agricultural University, Beijing 100094, China; 2 Veterinary Pathobiology, Texas A&M University and Texas Agricultural Experiment Station, College Station, TX, 77843-4467, USA

## Abstract

**Background:**

Random gene inactivation used to identify cellular functions associated with virulence and survival of *Brucella *spp has relied heavily upon the use of the transposon Tn5 that integrates at G/C base pairs. Transposons of the *mariner *family do not require species-specific host factors for efficient transposition, integrate nonspecifically at T/A base pairs, and, at a minimum, provide an alternative approach for gene discovery. In this study, plasmid vector pSC189, containing both the hyperactive transposase C9 and transposon terminal inverted repeats flanking a kanamycin resistance gene, were used to deliver *Himar1 *transposable element into the *B. melitensis *genome. Conjugation was performed efficiently and rapidly in less than one generation in order to minimize the formation of siblings while assuring the highest level of genome coverage.

**Results:**

Although previously identified groups or classes of genes required for virulence and survival were represented in the screen, additional novel identifications were revealed and may be attributable to the difference in insertion sequence biases of the two transposons. Mutants identified using a fluorescence-based macrophage screen were further evaluated using gentamicin-based protection assay in macrophages, survival in the mouse splenic clearance model and growth *in vitro *to identify mutants with reduced growth rates.

**Conclusion:**

The identification of novel genes within previously described groups was expected, and nearly two-thirds of the 95 genes had not been previously reported as contributing to survival and virulence using random Tn5-based mutagenesis. The results of this work provide added insight with regard to the regulatory elements, nutritional demands and mechanisms required for efficient intracellular growth and survival of the organism.

## Background

Previous studies designed to identify genes related to *Brucella *virulence and survival utilized Tn5-based mutagenesis strategies. Although useful in identifying numerous factors, this approach may be restricted in potential by the sequence bias for integration of Tn5 in GC-rich DNA sequences [1]. In contrast, *Himar1 *transposable element has a different target specificity and integrates at TA base pairs [2]. As a result of these different specificities, the two transposons are expected to provide complementary tools for gene discovery related to virulence and intracellular survival. However, rather than identifying completely new classes of genes, the expectation was that additional genes within previously described groups would be identified.

Functional characterization and sequence analysis of the *Brucella *genome have identified few classically defined virulence genes. When narrowly defined, virulence factors are those functions specifically designed to interact with the host cell, to enhance replication or survival, and have no effect on the organism when grown under laboratory conditions. Although this concept is useful when attempting to restrict the number of mutants and to target functions or mechanisms that are specific for survival of the organism, it may overlook the significance of gene products that sustain the organism in crucial intracellular environments. *Brucella *virulence results from the ability of the organism to survive and replicate within the professional phagocytic cells of the host [3]. Similarities between *Brucella *and plant pathogens are notable in the conservation of metabolic functions with the potential to enhance survival and persistence within different environments [4]. Much has been made of the environment in which *Brucella *sp. persist and replicate as well as the importance of several metabolic pathways to survival [5-8]. Perhaps more importantly, some metabolic pathways could provide specific targets for the development of new antibiotics capable of restricting intracellular replication. However, physical characterization of the "brucellasome" is just beginning [9,10] and it is important to remember that mutants exhibiting reduced *in vivo *replication may reflect minor growth defects that are exacerbated in the host as a result of exposure to additional stress. As such, it is difficult to make general predictions concerning nutritional pathways and relative importance to survival and virulence.

The work reported extends knowledge of pathogenic and metabolic functions necessary for survival and helps to improve definition of the replicative niche in which fully virulent *Brucella *persist. Intracellular survival in macrophage is responsible for virulence in the host, and in the experiments described, macrophage were used to screen a bank of mutants for defects in intracellular survival. The mouse provides a confirmation of virulence potential and is used here as a model for other animal species. In this model, the organism replicates and colonizes the spleen in elevated numbers causing a splenomegaly, but over time the numbers of organisms decline and splenomegaly subsides (a property that has not been characterized in other species including man). The outcome of these experiments is the identification of a large number of genes associated with virulence, of which nearly two-thirds have not been identified using random insertion mutagenesis.

## Results

### Construction of the mariner mutant bank

pSC189, containing the *Himar1 *transposable element in *E. coli *β2155, was the kind gift of Eric Rubin (Harvard School of Public Health). Following conjugation, as described in Materials and Methods, the viability of *Brucella*, as determined by the overall number of colony forming units (CFUs) recovered following conjugation, was shown to decrease 10–20% over the course of the experiment while the number of *E. coli *β2155 CFUs increased as much as fourfold. Under these conditions transformation of *Brucella *to kanamycin resistance occurred at a frequency of 1 in 100 to 1000 *Brucella*, and transformants were isolated by plating on TSA supplemented with kanamycin. The generation time was determined to be four hours under these conditions, and few if any siblings were expected to be identified. Digestion of genomic DNA with NcoI to produce two characteristic fragments following Southern blotting revealed that each of 100 mutants selected at random had inserted into different regions of the genome and that selection provided mutants with unique transposon insertions (data not shown). Sequence analysis described below is also consistent with a low number of siblings in the mutant bank.

### Identification of mutants with reduced survival relative to parental strain in macrophages

**Figure 1:**
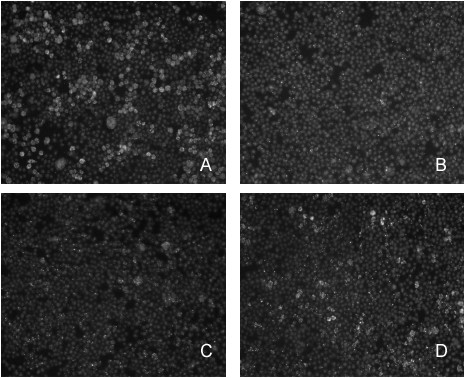
**Fluorescence screen of *Himar *1 mutant replication in macrophage**. Preliminary screening was performed using J774.A1 macrophage-like cells that were infected with *B. melitensis *16 M (panel A) or individual mutants (panels B, C, D) in 96-well microtiter dishes at MOI of 50, as described in Materials and Methods. Replication of each mutant was visualized using goat anti-*B. melitensis *and donkey-anti-goat IgG-Alexa Fluor 488. Images similar to those shown were captured using an Olympus IX70 inverted fluorescent microscope.

To identify mutants with reduced capacity to survive and replicate intracellularly, all the mutants were analyzed using the mouse macrophage-like cell line J774.A1 in the 96-well tissue culture plates. In order to minimize the number of false positives attributable to poor growth of the inoculum, mutant growth was determined after 48 hours *in vitro *by optical density readings using a BioRad microplate reader; mutants exhibiting poor growth in liquid media were eliminated from analysis. Cells were infected at an approximate multiplicity of infection (MOI) of 50 in duplicate, and after 48 hours intracellular bacteria were stained with goat anti-*B. melitensis *16 M antibody and Alexa Fluor 488-conjugated donkey anti-goat IgG (Molecular Probes, Eugene, Oregon, USA) to avoid artifacts due to over expression of green fluorescent protein (GFP) [11]. Following the first round of fluorescent microscopic examination, 1,020 mutants (5.5% of the mutant bank) were identified as attenuated based on limited intracellular replication or a reduced number of infected cells (Fig. 1). A second round of fluorescent microscopic evaluation was performed with the 1,020 mutants to eliminate mutants that were mischaracterized (i.e., false positives) in the first round of screening. Following this second round of screening, 443 mutants (2.4% of the mutant bank) were confirmed via fluorescence microscopy as attenuated for intracellular growth.

In order to better quantify the attenuated phenotype of these mutants, replication was monitored relative to the parental strain *B. melitensis *16 M over a 48 hour period in macrophages using the gentamicin protection assay described in Materials and Methods. Mutants exhibiting reduced recovery (tenfold or lower CFU compared to the parental strain) after 48 hours of growth in macrophage were selected for DNA sequencing (n = 200). These mutants were thought to encode critical regulatory functions or pathogenic mechanisms that more drastically affect survival. The remaining 243 mutants were not characterized in this study. DNA sequencing of the 200 mutants revealed the interruption of only 95 genes (Table 1), and 36 of the 95 genes identified were interrupted in multiple mutants. However, insertions in only two genes appeared to have identical transposon interruptions and to represent potential siblings consistent with earlier claims of elevated recovery of independent interruptions. Several publications have reported the identification of genes involved in virulence and survival of *Brucella *using Tn5-based random mutagenesis [6,12-14]. Yet, the use of the mariner transposon has revealed 57 novel genes insertions out of 95 identified in the studies reported here that have not been identified in any of those previous studies (Table 1).

Novel gene interruptions (and some previously characterized mutations) were further evaluated using the macrophage survival assay and the mouse splenic clearance model of infection. The results of this analysis are provided in Table 2. The identified virulence genes/operons are distributed evenly on the two chromosomes with clustering pronounced in *virB*, *luxR*, *gltAB *and *pur *loci (Table 2). Functions may be ascribed to 49 of the 57 genes; these are summarized in Table 2 in which the genes identified are classified according to Clusters of Orthogonal Groups (COGs). Seven genes lacked orthologs within microbial databases, and at least five others require additional characterization to confirm function. In addition, ten of fifty-seven newly identified interruptions are located within intergenic regions (indicated by an "/") between previously untargeted genes and will require additional characterization to confirm which of the flanking genes is responsible for attenuation of survival.

### Confirmation of reduced survival relative to parental strain in the mouse splenic clearance model

**Figure 2:**
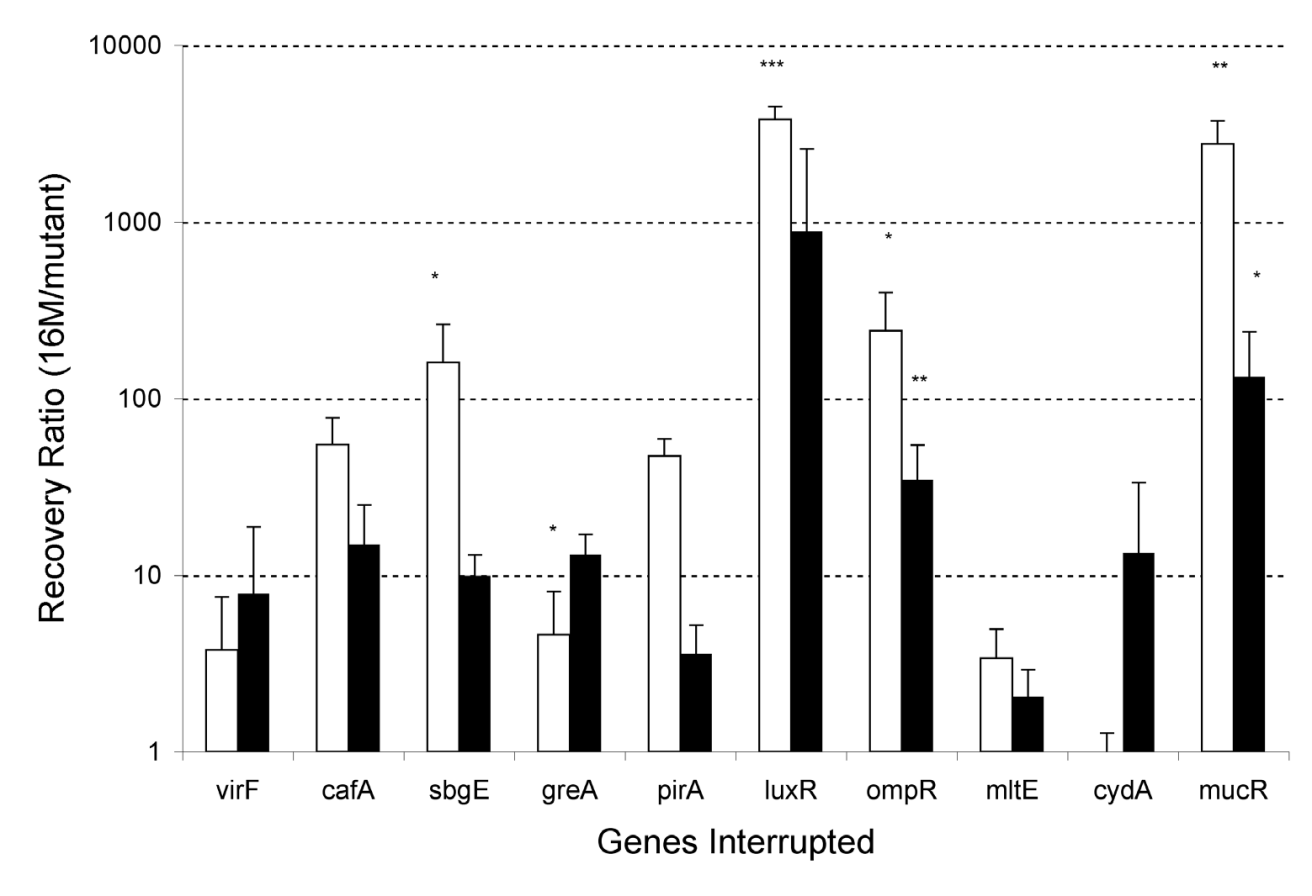
**Survival characteristics of novel and previously identified mutants validated in the mouse splenic clearance model of infection**. To confirm attenuated virulence several of the mutants were evaluated for survival in the mouse splenic clearance model using a 1:1 mixture of parental wild-type (*B. melitensis *16 M) and mutant to inoculate mice (n = 10 per group) with an overall dose of 5 × 10^6 ^cfu. At one (white bars) and four weeks (black bars) post inoculation spleens were excised from the mice, and recovery of the *B. melitensis *16 M and the mutant were determined as described in the Materials and Methods. Recovery of *Brucella *is presented as a Survival Ratio (CFU 16 M/CFU mutant) corrected for variation in the input ratio in the inoculum calculated the same way.

To confirm the relationship between reduced survival in macrophages and rapid clearance from the host, a subgroup of 22 newly identified mutants were evaluated for survival in the mouse splenic clearance model. The results confirm the predictions based on reduced survival in macrophages and suggest previously unidentified key roles for these genes in survival in the animal model (Table 2 and Fig. 2). In contrast, mutants that exhibited normal intracellular growth patterns in the fluorescence assay did not exhibit significant differences in survival in the mouse model (data not shown). Among the mutants exhibiting extremely of reduced survival were several with *Himar *insertions in important regulatory elements. Although not a novel identification, interruption of *luxR *(BME1116) severely reduced survival, while at least 4 other *luxR *orthologs present in the *Brucella *genome were not detected in this screen. Additional transcriptional regulatory elements that appear to be involved in regulating virulence in *Brucella *are *virF *(BMEI0371), sigma factor; *ompR* (BMEI0066), a two component response regulator; *rpoZ* (BME1297), RNA polymerase omega subunit and transcriptional regulatory elements *mucR *(BME1364), *lysR *(BMEI0513) and *deoR *(BME0304). Additional factors important for survival include *virB*, LPS biosynthesis genes and several metabolic functions. Differences were observed in the survival ratios over time for several of the mutants and may reflect differences in the timing of their expression. For example, genes encoding cytochrome *bd *oxidase function appear to be important later in infection, while most other functions have a more dramatic effect early in infection (Fig. 2).

### *In vitro *growth characteristics of attenuated mutants

An issue raised in earlier work with regard to the identification of virulence genes is whether reduced survival observed in macrophages can be said to be specific to the intracellular environment. Although such a definition is meant to enhance focus on pathogenic mechanisms, it has the disadvantage of categorically eliminating mutants that may be useful either in characterizing the intracellular environment in which *Brucella *persists or for vaccine development. Mutants exhibiting obvious growth defects *in vitro *were naturally eliminated during early steps of the screening process due to poor growth. However, mutants exhibiting modest defects were present among the group identified. For example, purine auxotrophs appear to grow poorly *in vitro *when in competition with the parental wild-type organisms (Table 2). Yet, their growth defect in macrophages reveals an interesting and important aspect of the environment in which the organism persists within the cell [15]. For all of these reasons we have provided the competitive growth ratios for comparison with macrophage and mouse splenic clearance (Table 2). Overall the *in vitro *growth ratios were smaller than those observed in mice or in macrophages, although the latter was not performed under competitive growth conditions but reflected relative macrophage growth potential of mutant and wild-type. We have assumed, but not established with certainty, that small growth defects are exacerbated when the organism is exposed to stressful conditions within the cell. Although several of the mutants identified may be so described, reduced survival could be claimed for mutants exhibiting the slightest alteration of growth *in vitro*, and for that reason, should not be used as sole criterion to eliminate mutants from careful consideration.

**Figure 3:**
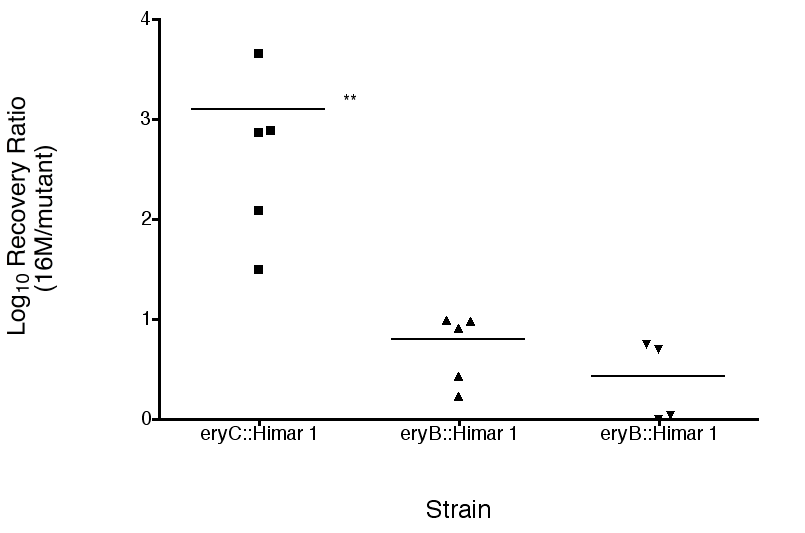
***Himar *interruption of the erythritol locus has varying effect on virulence**. The virulence of mutants interrupted in different genes of the erythritol locus were evaluated in the mouse splenic clearance model using mixtures (approximately 1:1) of *B. melitensis *16 M and individual mutants containing interruptions in either *eryB *or *eryC *as indicated. Recovery of the parental wild-type and mutant were determined one week after infection by plating spleen homogenate on TSA with and without kanamycin as described in Materials and Methods. The ratio of wild-type to mutant recovered is corrected for variation in the input ratio and the final value is expressed as a ratio of 16 M to mutant recovered as described in Materials and Methods. The values shown reflect the ratios recovered plotted on a log scale. Differences in the average values were evaluated for significance via one-way ANOVA with Kruskal-Wallis test followed by Dunn's multiple comparisons between all groups p-value <0.0045 (**).

Extrapolations based on the interruption of a single gene within a metabolic pathway should be carefully proposed, as illustrated by the difference in survival characteristics reported previously [8,16] and confirmed here for interruptions in *eryC *and *eryB *in *B. melitensis *(Fig. 3). The mouse splenic clearance reveals a tenfold difference in survival characteristics of *eryC *and two independently derived *eryB *mutants despite similar sensitivities to erythritol (Fig. 3 and data not shown) confirming previous results evaluating *B. suis *and *B. abortus *[8,16]. Since the metabolic utilization of erythritol should have a similar effect on growth of the organism, differences in survival may be explained by differences in the buildup of toxic metabolic intermediates.

### Genes that were not identified using *Himar transposable *element insertion mutagenesis

Published reports suggest the potential identification of genomic islands (GI) that may explain the gain or loss of virulence function in *Brucella *[17]. Examination of the mutants identified in this work revealed an insertion in only one of the 141 loci representing the 9 GI described. This low level agreement is quite striking and is not attributable to differences in AT content of the GIs and the *Brucella *genome. Interestingly, despite the near saturated mutagenesis of the operons encoding the T4SS and the purine metabolic pathway, there were no hits in any gene within the flagellar locus (BMEII1078–1089), which is thought to potentially encode a type 3 secretion system (T3SS). However, interruption was identified in at least one neighboring locus (BMEII1095).

## Discussion

The genomes of *Brucella *species are replete with genes encoding metabolic functions but contain few that encode classically defined virulence genes. Previous studies have reported the significance of metabolic functions to survival but have not claimed saturation mutagenesis of the *Brucella *genome. This leaves room for the identification of additional functions [5-7,12]. The aim of this study was to identify additional functions required for virulence using a transposon with different target specificity (ts) and elevated transposition frequency. The reported results confirm these differences and identify a number of genes that were not previously identified using a random approach. The genes identified as important for intracellular survival in this work have been arranged according to their COGs (Table 1). Reviews of several databases have revealed discrepancies in overall COG assignment, and in the analysis shown, the genes are distributed according to COG assignments in the Integrated Microbial Genomes database (DOE).

Analysis reveals an elevated propensity for interruption of genes involved in intracellular trafficking (U), nucleotide metabolism (F), transcription (K), membrane biogenesis (M) and energy production and conversion (C). This frequency is determined by calculating the number of genes assigned to each COG divided by the total number of genes identified and ignores the overall frequency with which a particular gene or group of genes was identified in the screen. In an alternative calculation, the total number of independent hits within a gene, identified by unique insertion point via DNA sequence analysis, was also calculated as a fraction of the total number of transposon insertions. When this number is divided by the fraction of the genome represented by a COG number, results reflect the relative specificity with which a particular COG is identified (referred to here as target specificity (ts)). For example, a target specificity of 1.0 indicates that the frequency of *Himar transposable element 1 *insertion in a particular COG is identical to the frequency genes of that COG appear in the genome. Values of ts less than 1.0 suggest that genes represented by these COGs are of reduced importance for intracellular survival. Target specificity values much greater than 1.0 are consistent with enhanced identification or importance for intracellular survival. Using this approach, the COG with the highest value (≥ 13.3) encoded functions associated with intracellular trafficking, secretion and vesicular transport. Most notable in this group are the genes encoding the type IV secretion system for which 47 separate insertions were identified in 10 of 11 *virB *genes in this study.

The next most frequent group identified included the genes encoding nucleotide transport and metabolism functions (ts ≥ 3.5). This group of mutants has also been previously described, and the results here confirm the importance of this biosynthetic pathway with 18 separate insertions in genes encoding nine distinct enzymatic functions that attenuate survival [15]. The failure of *Himar transposable element 1 *mutagenesis to identify a single locus involved in pyrimidine biosynthesis may suggest that saturation has not been achieved or that transposon insertion site specificity may be responsible. However, analysis of the overall AT content of these loci does not support the latter argument. Pyrimidine requiring auxotrophs may have been eliminated from consideration, along with many other mutations, as a result of a moderate reduction in survival, i.e., tenfold or less. The poor competitive growth of purine mutants *in vitro *appears to explain their poor growth in macrophage and indicates a low level of free nucleotides within the intracellular milieu. Differences in relative survival rates of pyrimidine and purine mutants cannot be explained.

In contrast with the elevated ts value for nucleotide biosynthesis, the values for amino acid and carbohydrate metabolism are low, ≤ 0.97 and 0.15, respectively. Although the value for amino acid biosynthesis appears equivalent to the distribution in the genome overall, at 1.0, the number is greatly reduced (ts = 0.10), if insertions in glutamate synthase genes, *gltA *and *gltB*, representing 21 out of 23 mutants identified, are excluded. This suggests that amino acid metabolism has a limited effect on overall survival. Previous studies have reflected on the potential for poor nutritional content of the *Brucella*-containing vacuole (BCV) based on the interruption of several important biosynthetic pathways [7]. Although the results confirm the importance of purine nucleotide metabolism for survival, the paucity of interruptions in genes concerned with amino acid metabolism was unexpected. The poor survival of *Brucella gltBD *mutants is consistent with the concept of a *Brucella *containing vacuole that is reduced in nutritional content [5-7], but the role of glutamate synthase in nitrogen metabolism may be even more critical. The identification of at least one peptide transport system (BMEII0285) among many located on genomic islands may preclude the need to synthesize amino acids explaining the poor recovery of mutants defective in amino acid biosynthesis [15,17]. The limited number of interruptions within the genomic islands (1 out of 141 genetic loci) may be explained by redundancies in transport functions. However, caution must be used in these early interpretations, since *gltBD *has also been shown to alter the osmotic sensitivity of bacteria that could play a crucial role during intracellular survival [18]. Evidence from closely related Rhizobial species also indicates the ability of *gltBD *mutants to colonize root nodules by these mutants despite an inability to fix nitrogen, and in this case, it has also been rationalized that the plants provide nitrogen in the form of amino acids [19].

Additional activities associated with amino acid metabolism include two proteins that regulate glutamine synthesis associated with nitrogen metabolism; *glnD*, the primary sensor of nitrogen status, and *glnE*, a protein that regulates glutamine synthase activity through covalent adenylation [20]. Clearly the assimilation of nitrogen is important to the survival of the organism and glutamate synthase appears to be a critical control point for survival.

Among the mutants attenuated for virulence, a large number were interrupted in genes required for lipopolysaccharide biosynthesis (ts ≥ 1.9). All of the genes identified (n = 15) have a direct role in the biosynthesis and assembly of lipopolysaccharide or the cell wall to which the LPS is attached. Nearly half of these contained interruptions within the previously described O-antigen biosynthetic locus (BMEI1390–1427) [21]. Several others were associated with cell wall metabolism that also caused a rough phenotype; this would explain reduced survival relative to parental strain. An additional locus involved in O-antigen expression also represents a basic metabolic function (BMEII0899) encoding phosphomannomutase [22]. Yet, a second *pmm *identified in the genome (BMEI1396) and within the main O-antigen biosynthetic locus shares up to 55% identity with BMEII0899 but fails to function in the place of BMEII0899 indicating either regulation of expression only under specific conditions or divergence of function.

Two other novel mutants affecting O-antigen production interrupt genes encoding soluble lytic murein transglycosylases (BMEI1302). Although it is interesting to speculate concerning the potential role in enhancing bacterial virulence based on similarities with plant pathogens [23], the rough phenotype of these mutants alone may be sufficient to account for their attenuated virulence. Of the remaining 50 novel mutants, all appear to make full-length LPS based on agglutination tests [24]; however, the effect of these various mutations on the production of other known virulence factors, cyclic-β-glucan (CβG) and VirB proteins, remains to be determined.

Defense mechanisms (V) and energy production and conversion (C) genes are grouped together in this discussion based on the demonstrated requirement for genes of the latter group, most notably the requirement for the previously identified alternative terminal ubiquinol oxidase complex, *cyd*DCBA (BMEII0759–62) in resistance to oxidative stress [25]. Novel identifications include the oxidase/reductase enzymes glutaredoxin (BMEII0932) and glutathione reductase (BMEI0972) that, in addition to relieving stress, may prove invaluable in the synthesis of ribonucleotides including ATP and underscore the importance of purine metabolism [26]. Also among energy production and conversion (C) functions, we have identified glycerol-3-phosphate dehydrogenase (BMEI1749) and genes encoding erythritol utilization functions (BMEII0428 and 429).

Although mutation in the erythritol pathway has been previously described, additional discussion is warranted [8]. In recent reports, interruptions of both genes in *B. suis and B. abortus *were described as attenuating survival independently of erythritol utilization consistent with the concept that toxic intermediates are responsible for reduced survival [8,15]. Experiments reported here revealed a more severe attenuation of *eryC::Himar *mutants when a competitive infection model was employed (Fig. 3 and Table 2). The observation that *eryC*::*Himar *attenuates survival more drastically than *eryB::Himar *mutation suggests the toxic nature of the product of D-erythrulose 4-phosphate dehydrogenase (EryC), and the observation that erythritol tolerance resulting from reduced erythritol uptake improves survival is consistent with this interpretation [8]. Perhaps most importantly, it is prudent to stress that these results suggest that erythritol is not an essential carbon source in the systems employed; otherwise, mutation in either gene would be expected to prevent bacterial growth and limit virulence. Furthermore, similar situations may exist in other operons indicating the need to carefully examine defects in multiple functions before concluding that the entire pathway is essential for survival.

A single defense mechanism (V) was identified, the ortholog of the multidrug resistance locus *emr*A (BMEI0926) capable of preventing the buildup of toxic by-products. The linkage between the reduction of the oxidation potential within the bacterium and energy efficiency are both presumably critical contributions to survival. Several additional functions were identified that are important in preserving DNA fidelity and the efficiency of gene expression under stressful conditions that may otherwise lead to DNA damage.

Genes associated with translation, ribosome structure and biogenesis (J) include ribosomal large subunit pseudouridine synthase C (BMEI0983) and ribonuclease E/Zn metalloprotease (BMEI1057). Both may control organism survival via their roles in RNA metabolism [27,28]. The interruption between Rnase PH and HrcA (heat shock protein repressor) (BMEI1775–6) may affect tRNA processing, the addition of polyA tails to mRNA regulation of expression from heat shock loci depending on polarity [29-32].

Among genes encoding functions associated with posttranslational modification, protein turnover and chaperones (O), was the ATP-dependent clp protease ATP-binding subunit clp required to relieve stress, reportedly more important for growth of mice than in macrophage [33,34]. Only a single gene associated with coenzyme transport and metabolism (H) was identified in this screen, the TonB-dependent metal chelator (BMEI0657) [35]. Several genes were identified within the general function prediction group (R); the glycine cleavage T protein (aminomethyltransferase) is part of the previously described *gcv *operon reportedly required late in infection [12]. The metal-dependent hydrolase (β-lactamase) (BMEI1143) has been shown to function as possible AHL-lactonase [36]; Pirin (BMEI1499) induced in cyanobacteria under conditions of stress appears to function in the transcription of other genes [37]; and florfenicol resistance protein (BMEI1867) is frequently identified with multi-drug resistant bacteria in cattle [38,39].

Of special interest in this work was the identification of a number of novel genetic loci that regulate gene expression essential to intracellular survival. This group includes both functions associated with signal transduction (ts = 2.4) and transcriptional control (ts = 2.1). Of these *muc*R, *tet*R, *lux*R and *hyd*G stand out as most critical for survival. The importance of LuxR (BMEII1116) and HydG (BMEII0011) in survival has been previously evaluated, but the other regulators (MucR, TetR, OmpR and VirF) have not. TetR reportedly had no effect on survival in a systematic approach study [40], and the fact that the transposon insertion identified was actually intergenic suggests a possible influence on *luxR *expression (BMEII1116) rather than from *tetR *(BMEII1117). TetR regulators have been observed in a number of bacteria and are frequently involved in the regulation of quorum sensing loci [40,41]. MucR regulators are frequently observed among the α-proteobacteria in which they regulate expression of exopolysaccharide production [42]. Preliminary results reveal that this mutant makes normal levels of LPS (O-antigen) and VirB gene products. The basis for its reduced survival relative to parental strain is currently under investigation. OmpR represents a two-component response system similar to the previously described BvrR/BvrS system [43]. VirF is a sigma-70 homolog with greatest conservation in regions 2 and 4 involved in -10 and -35 region recognition and binding [44], and *rpoZ *encodes an RNA polymerase omega subunit important for assembly and function of RNA polymerase. However, the proximity of this locus to the BMEI1296, which was also identified in this screen and recently described in more detail, suggests that more careful analysis of nonpolar deletions may be required to confirm the role of the *rpoZ *locus in virulence [45]. The cold shock protein encoded by *csp*A may also be a critical factor, although the intergenic nature of this insertion and others suggests a possible role for small RNAs in survival and virulence and such analysis is in progress. GreA is required to prevent pausing transcription elongation at specific sites and may reduce the survival of the organism under stringent growth conditions as revealed during competitive *in vitro *growth.

## Conclusion

Overall, more than half of the genes identified in this study using a random mutagenesis approach have not been identified with virulence of *Brucella*. However, in many cases the genes identified are associated with well-known groups of functions affecting survival of *Brucella*. Among these are the genes associated with the synthesis of the T4SS, LPS biosynthesis and purine metabolism. The failure to previously identify these mutants may either be attributable to the difference in insertion site specificity of the mariner transposon relative to Tn5 or the potential that the Tn5 screens performed to date have not achieved genome saturation [15,46]. In defense of previous work, the stated goal was the identification of genes required for virulence/survival and there was no claim that saturated mutagenesis was achieved. Similarly, the work reported here still cannot claim with certainty that saturation mutagenesis has been achieved. In addition, the potential for both false negative or false positive results cannot be eliminated. Therefore, it is still important to verify the contribution of each gene identified by performing both deletion mutagenesis and complementation of the genes in question. This work is currently in progress focusing on the novel identifications reported. Although a handful of the genes identified were shown to cause *in vitro *growth defects in rich media, only a few of these exhibited growth defects as low as that observed in macrophages or in the mouse splenic clearance model. Interestingly, a number of mutants exhibiting reduced *in vitro *growth have been previously described as attenuated for intracellular survival [6,15]. Again, it should be noted that although those functions were identified as important for intracellular survival, they were not described as specific to intracellular survival. These results emphasize the need to examine these effects on a gene-by-gene basis and argue against broad generalizations concerning reduced fitness vs. pathogenesis. However, the use of such defects in the construction of vaccine strains remains a viable option, since such organisms may be easily prepared using supplemented media while retaining *in vivo *growth defect.

## Methods

### Bacterial Strains and Media

*Brucella melitensis *16 M (American Type Culture Collection 23444) re-isolated from an aborted goat fetus and all *Himar1 *transposable element-derived mutants were routinely grown in tryptic soy broth (TSB) or tryptic soy agar (TSA) with/without kanamycin at 37°C. Bacteria from liquid culture or resuspended plate cultures are quantified spectrophotometrically using a standardized growth curve. Accurate determinations of culture density are determined by serial dilution and plating portions on TSA plates. For conjugation, *Escherichia coli *β2155 donor strain bearing the plasmid pSC189 and containing a *Himar1* transposable element was cultivated in TSA containing 50 μg/ml DAP (diaminopamelic acid) [47]. Both parental bacterial strains and their mutated derivatives were maintained as frozen stocks following the addition of glycerol to a final concentration of 25%(v/v). Antibiotics were used to supplement the media at final concentrations of 100 μg/ml (kanamycin) and 40 μg/ml (gentamicin).

### Conjugation and mutagenesis

*B. melitensis *16 M was grown on solid media up to 72 hours as described above. *E. coli *β2155 with or without pSC189 was grown on TSA supplemented with DAP (50 μg/ml) and kanamycin for 24 hours. Donor and recipient bacteria on each of the plates were harvested in 5 ml of peptone-saline [1% (w/v) Bacto-peptone™ and 0.5% (w/v) NaCl] containing DAP. Equal volumes (100 μl) of bacterial suspensions were mixed together to provide a donor to recipient ratio of approximately 1:100. The suspension was plated on nitrocellulose filters placed on the surface of TSA plates supplemented with DAP (50 μg/ml) and incubated for no more than two hours at 37°C. Serial dilutions of the conjugation mixtures were prepared in peptone saline and plated onto TSA plates supplemented with kanamycin (100 μg/ml) to evaluate *Brucella *transformation efficiency. Using these conditions, growth of the donor *E. coli *strain β2155 strain was repressed by the absence of DAP, and no exconjugants were obtained in mixtures of *B. melitensis *16 M with *E. coli *β2155 lacking pSC189. The remaining bacterial conjugation mixture was stored in peptone saline at 4°C. The transconjugants were plated on TSA containing 100 μg/ml kanamycin and the isolated colonies were transferred to 96-well plates containing TSB supplemented with kanamycin. The bank consists of 18,708 mutants in 195 microplates and represents sixfold coverage of the genome (i.e., averages 6 insertions per gene) with greater than 99% assurance of covering the entire genome [48].

### Intracellular survival

The method used has been described extensively elsewhere [49]. Briefly, *Brucella *were grown in TSB or TSB with appropriate antibiotic for approximately 24 hours. J774.A1 macrophage at low passage were used to seed the wells of a 24 well plate at 2.5 × 10^5 ^per well in 0.5 ml DMEM. Bacterial cultures were washed and diluted 5-fold with PBS prior to addition to each of four wells for each strain at a final MOI (multiplicity of infection) of 50 determined as described above. The plates were centrifuged at 200 × g for 5 min at room temperature and then incubated at 37°C for 20 minutes. The infected cell monolayers were washed with PBS (or peptone saline) three times, overlaid with 0.5 ml of DMEM containing 50 μg/ml gentamicin and incubated at 37°C for various times. Following these incubations the media was replaced with 0.5 ml of solution containing 0.5% (v/v) Tween-20 to lyse the macrophage monolayer. The cell lysate was pipetted vigorously to ensure cell lysis and serial dilutions were prepared in PBS and portions plated on TSA supplemented with antibiotic as necessary to evaluate bacterial survival. The results are presented as the difference in replication for wild-type and mutant strains using the following formula: log_10 _[(CFU 16 M at 48 h/CFU 16 M at 1 h)] minus log_10 _[(CFU mutant at 48 h/CFU mutant at 1 h)]. The values presented represent the means of at least three separate experiments.

### Identification of attenuated mutants

Based on the calculated transformation efficiency, conjugation mixtures were diluted and plated onto TSA supplemented with kanamycin. Single colonies were visible within 72 hours and transferred from the TSA plates to individual wells of 96-well microtiter dishes containing TSB supplemented with kanamycin and incubated for 48 hours at 37°C. These dishes were used to prepare duplicate dishes for storage at -80°C after adjusting the cell suspensions to 20% (v/v) with sterile glycerol and were also used to prepare fresh culture for macrophage infection following resuspension (1:20 dilution in 100 μl) in fresh TSB supplemented with kanamycin and incubation at 37°C for 24 hours. Murine macrophage-like J774.A1 cells were seeded in 96-well microtiter dishes at 5×10^4 ^cells/well and incubated for 18 hours at 37°C in atmosphere containing 5% (v/v) CO_2_. Infections were performed with the addition of 10 μl of the 24-hour cultures of bacteria at a final multiplicity of infection (MOI) of approximately 50. Bacteria were pelleted onto the cell monolayers by centrifugation for 5 minutes at 200 × g to synchronize uptake. Cultures were incubated at 37°C for 20 minutes prior to removal of extracellular bacteria by washing with peptone saline. Incubation was continued at 37°C up to 48 hours following the addition of 200 μl complete DMEM supplemented with 40 μg/ml gentamicin.

Following incubation, the media was removed and the monolayer fixed with 200 μl of 3.7% (v/v) formaldehyde at room temperature for 30 minutes. The fixed monolayers were washed three times with PBS [10 mM sodium phosphate and 150 mM NaCl, pH 7.4], followed by incubation with 50 μl of goat-anti-*B. melitensis *16Mserum diluted 1:500 in PBS-TT [PBS with 0.05% (w/v) Tween-20 and 0.05% (v/v) Triton X-100] prior to incubation for 1 hour at room temperature. The primary antibody was removed and the plates washed three times with 200 μl PBS-T (PBS with 0.05% (v/v) Tween 20). Fifty μl of donkey-anti-goat IgG-Alexa Fluor 488 diluted 1:500 in PBS-TT was then added to each well and incubation continued for 1 hour at room temperature in the dark. The plates were washed again three times with PBS-T followed by PBS and evaluated via fluorescence microscopy (Olympus IX70) as described previously [49]. In contrast to infection by *B. melitensis *16 M that grows well and exhibits numerous cells full of bacteria, multiplication of attenuated mutants is greatly reduced in number and/or present in only a few macrophages.

### Isolation of genomic DNA and identification of interrupted loci

Extraction, preparation and subsequent DNA sequence analysis were all performed as previously described with the following specific changes warranted by the use of mariner transposon *Himar 1 *[12]. First, genomic DNA from attenuated mutants was digested with HaeIII. Amplification of the interrupted loci utilized mariner-specific forward 5'-CAACACTCAACCCTATCTCG-3' and reverse primers 5'-CACTCAACCCTATCTCGGTC-3' under the following conditions: 95°C 4 min, 30X (95°C 30 sec, 57°C 30 sec, 72°C 90 sec), 72°C 7 min. PCR products were purified from agrose gel using the QIAquick Gel Purification Kit (Qiagen, Valencia, CA). Sequencing was performed using the primer 5'-CACTCAACCCTATCTCGGTC-3' at the Gene Technologies Laboratory (GTL, Institute of Developmental and Molecular Biology, BSBW 437 Texas A&M University, College Station, TX 77843-3155).

The sequences were blasted against the *B. melitensis *genome database [50] to identify and confirm the identity of the disrupted genes. Gene function predictions and assignment to clusters of orthologous genes (GOGs) was performed using the Integrated Microbial Genomes database available through the Department of Energy (DOE) web page [51]. Additional analysis, including identification of the site of transposon insertion were performed using MacVector™ (Accelrys, Inc.) using the sequence for *B. melitensis *16 M generated by DelVecchio et al [52]. Locus tags for the large chromosome BMEI (1–2060) and for the small chromosome BMEII (1–1138).

### Southern blotting

Plasmid pKD4 was used as template to provide the probe used for Southern blotting, which was prepared by PCR amplification of the neomycin phosphotransferase gene (*nptII *gene) using forward 5'-CGGGATCCCGCACGTCTTGAGCGATTGTGTAGG-3') and reverse primers 5"-CGGGATCCCGGGACAACAAGCCAGGGATGTAAC-3' [53]. Cycling conditions were 95°C for 5 minutes, 30 cycles of 95°C 30 seconds/62°C 30 seconds, 72°C 1.5 minutes and a final extension at 72°C for 10 minutes. PCR products were purified using the QIAquick Gel Extraction Kit (Qiagen, Valencia, CA). These PCR products were labeled with fluoroscein, as described by the manufacturer (NEN Life Science), and the concentration of the labeled probe was used at a range of 10–20 ng/ml. Restriction digestion, agarose gel electrophoresis of restriction digested genomic DNA, Southern blotting, probe preparation, hybridization and film exposure were all as previously described [49]. Two enzymes (HindIII and NcoI) were used to evaluate the transposon insertions in the mutant genomes. There are no HindIII sites and one NcoI site within the transposon, resulting in a single and two *nptII *fragments, respectively.

### Survival in the mouse splenic clearance model

Survival of *B. melitensis *16 M and attenuated mutants in mice was performed as previously described [12]. Briefly a mixture (1:1) of parental strain and mutant was resuspended in PBS and inoculated into the peritoneal cavity of mice. Spleens were harvested as early as one week, and no later than five weeks, post inoculation and homogenized in PBS. Serial dilutions were prepared and the bacterial recovery determined by plating on TSA and TSA supplemented with kanamycin (TSAK). Wild-type and mutant will grow on TSA, while only the mutant exhibits growth on TSAK. The results are presented as the ratio of (CFU_wt_/CFU_mutant_) recovered to the ratio inoculated (CFU_wt_/CFU_mutant_) or the difference in log_10 _of these values and reflects the average of at least three separate assays.

### Competitive *in vitro *growth

*In vitro *and *in vivo *competitive growth and auxonography were performed; the competitive growth indices were calculated as described previously and reflect the median of at least three separate assays [12]. Competitive growth ratios are reported (as described above) for survival in the mouse splenic clearance model and are representative of at least three separate experiments.

### Statistical analysis

Primary screening in macrophage was performed twice for all 18,708 mutants, and only those exhibiting reduced survival in duplicate wells as described in the Results were chosen for additional analysis. Gentamicin protection assay was then performed for all 443 mutants, and those exhibiting tenfold or greater reduction in survival at 48 hours in this assay relative to 16 M were selected for further analysis. Additional gentamicin protection assays and survival in the mouse splenic clearance model were performed for selected mutants, and the survival ratios calculated as described above were analyzed using ANOVA via Prism™ with either Kruskal-Wallis or Freidman tests. Groups with statistically valid differences in their means were further evaluated using Dunn's multiple comparison post-test to establish differences from virulent organism (Graphpad, Inc.).

## Authors' contributions

QW carried out creation, cataloging and primary screening of the *Himar1 *transposable element mutant bank, identification of interrupted genes and drafting of the manuscript. JP participated in the creation and primary screening of the mutant bank, as well as sequencing and mouse splenic clearance model of infection. CT performed the survival analysis of the erythritol mutants *in vitro *and in macrophage. TAF conceived of the study, participated in its design and coordination, performed statistical analysis and finalized the manuscript.

## Figures and Tables

**Table 1:** *Himar *1 gene interruptions that attenuate survival of *B. melitensis *16 M in J774.A1 macrophages assorted according to COG‡

***Functional Class***	B. melitensis (BME) locus*	Genes Identified (#)	Fraction Identified (%)	Novel Genes (#)	Target Specificity^†^
Intracellular trafficking, secretion	II0025, II0026, II0027, II0028, II0029, **II0029/30 **II0030, II0032, II0033, **II0034**, **II0035**	11	12%	3	12.8
Nucleotide transport and metabolism	**I0233, I0295**, I0296, **I1123, I1124**, I1127, **I1204, **I1240, I1488, I1519	10	11%	5	3.5
Signal transduction	**I0066, **I1296, **I1327, I1807**, II0011	5	5%	3	2.4
Transcription	**I0304/5, I0371, I0508**, I0513, **I0513/4, I0731/2, I0808, I1178, I1297/8, I1364, I1647/8**, II1116, **II1116/7**	13	14%	11	2.1
Cell wall/membrane/envelope biogenesis	**I0498/9, **I0997, **I1302, **I1326, I1393, I1413, I1414, **I1415, I1416, **I1426, **I1427, **I1886, **II0380, II0472, **II0899	15	16%	7	1.9
Energy production and conversion	**I0972, I1749**, II0428, II0429, II0759, II0760, II0761, II0762	8	8%	2	1.2
Amino acid transport and metabolism	I0025, II0039, II0040, **II0285,**	4	4%	1	1.0
Replication, recombination and repair	I0040, **I0334, I1307, I2023, II0260**	5	5%	4	0.5
Translation, ribosome structure and biogenesis	**I0983, I1057, I1775/6**	3	3%	3	0.4
Function unknown	**I0186, I0193, I0490, I0540, I0603, I0732, II0045/6**	7	7%	7	0.4
Posttranslational modification, protein turnover, chaperones	**I0816, **I1804,**II0932**	3	3%	2	0.4
Defense mechanisms	**I0926**	1	1%	1	0.3
General function prediction	**I1143, I1282, I1487, I1499, I1867, I1894**	5	5%	5	0.2
Carbohydrate transport and metabolism	**I2031, **II1095	2	2%	1	0.2
Lipid transport and metabolism	I1553	1	1%		0.1
Coenzyme transport and metabolism	**I0657**	1	1%	1	0.1

‡ Genes assigned to COGs that are not listed were not identified.

* Numbers in bold reflect novel identification (n = 57). Those that are not bold have been identified previously using Tn5 by us and others [46]

/ Indicates intergenic insertion between two genes.

† ts or target specificity represents the ratio of insertions per COG divided by the fraction of the genome represented by that COG.

**Table 2:** Summary of survival characteristics of novel attenuated mutants

***Chr***	***BME***	***GENE FUNCTION***	***Gene***	***S v. R^a^***	***In vitro Survival Ratio***	***M∅ Survival Ratio^c^***	***Mouse Survival Ratio^c,d^***
Intracellular trafficking, secretion, and vesicular transport							
I	1894	Gramicidin S biosynthesis	grsT	S	-0.4	1.5	0.9
II	0029–30	VirB5-B6	virB5/6	S			
II	0034	Type IV secretion (channel protein)	virB10	S		2.5*	
II	0035	Type IV secretion (ATPase)	virB11	S		2.3***	
Nucleotide transport and metabolism							
I	0233	Phosphoribosylaminoimidazolecarboxamide formyltransfease	purH	S	0.4	0.9	
I	0295	Phosphoribosylaminoimidazole carboxylase ATPase subunit	purK	S	0.3	0.9	
I	1123	Phosphoribosylformylglycinamidine (FGAM) synthase	purS	S	0.9	2.9	
I	1124	Phosphoribosylformylglycinamidine (FGAM) synthase	purQ	S	1.9	2.2	
I	1204	Exopolyphosphatase (Ppx/GppA)	ppx	S	0.4	1.2	
Signal transduction mechanisms							
I	0066	Response regulator (two component)	ompR	S	0.6	1.3	1.9*
I	1327	Glutamate-ammonia-ligase adenylyltransferase (signal transduction)	glnE	S	-0.1	1.2	1.4
I	1807	Universal stress protein	uspA	S		0.4	0.1
Transcription							
I	0304/5	Hypothetical thioesterase-transcriptional regulator	deoR	S	-0.5	1.9	
I	0371	Regulatory factor (rpoE)	virF	S	-0.1	1.1	0.3
I	0508	Transcription elongation factor	greA	S	1.0	0.7	0.6
I	0513	Transcription factor	lysR	S		0.9	
I	0513/4	Transcription factor-hypothetical protein	lysR/hyp	S	0.2	0.7	
I	0731/2	Cold shock protein-hypothetical cytosolic protein	csp/hyp	S	0.2	1.1	0.9
I	0808	Transcriptional regulator (soxR)	merR	S	1.0	0.8	
I	1178	Transcriptional regulator (soxR)	merR	S	0.0		
I	1297/8	RNA polymerase omega subunit-hypothetical protein	rpoZ/hyp	S	0.2	1.3	
I	1364	Transcriptional regulatory protein	mucR	S	0.3	2.0***	3.0**
I	1647/8	Hyp-RegM (ATP-binding)	hyp/regM	S		1.0	-0.2
II	1116/7	Transcriptional activator LuxR-TetR families	luxR/tetR	S	0.1	1.6**	1.1
Cell wall/membrane/envelope biogenesis							
I	0498/9	Cold shock protein/soluble lytic transglycosylase	cspA/slt	R	0.0	1.7***	2.9
I	1302	Soluble lytic murein transglycosylase	mltE	R	0.6	0.7	0.4
I	1415	O-antigen export system permease protein	rfbD	R	0.1	1.3	
I	1416	O-antigen export system ATP-binding protein	rfbE	R	0.3	1.9**	
I	1427	UDP-N-acetylglucosamine 4,6-dehydratase	capD	R	-0.3	1.4	
II	0380	Acriflavin resistance protein A (membrane biogenesis)	acrA(hlyD)	S		3.4	
II	0472	Membrane fusion protein (membrane biogenesis)	mtrC(hlyD)	S	0.9	0.3	
Energy production and conversion							
I	0972	Glutathione reductase	gor	S	0.3	0.5	
I	1749	FAD-dependent glycerol phosphate dehydrogenase	glpD	S	0.2	0.7	
Amino acid transport and metabolism							
II	0285	Dipeptide transport system (inner membrane permease)	dppB	S		0.4	0.7
Replication, recombination and repair							
I	0334	AAA ATPase/DNA helicase/DNA damage checkpoint	ruvB	S	1.3	0.9	
I	1307	Integrase/recombinase (phage)	xerC (int)	S	0.1	1.1	
I	2023	ATP-dependent nuclease subunit A	uvrD/rep	S		0.9	
II	0260	GTP-binding protein	lepA	S	0.9	0.1	
I	1296	GTP-binding protein	lepA	S		1.0	
Translation, ribosome structure and biogenesis							
I	0983	Ribosomal large subunit pseudouridine synthase C	rluA	S	0.5	1.1	1.0
I	1057	Ribonuclease E/Zn metalloprotease	cafA	S	0.5	1.1	1.6
I	1775–6	Rnase PH-HrcA (heat shock protein repressor)	rph/hrcA	S	-0.2	1.0	0.6
Function unknown							
I	0186	Hypothetical cytosolic protein	dut	S	0.6	0.4	
I	0193	Hypothetical protein	hyp	S	0.4	2.4	1.5
I	0490	Hypothetical cytosolic protein	hycp	S	0.6	0.6	
I	0540	Hypothetical protein	hyp	S	0.0	0.6	
I	0603	Hypothetical protein	hyp	S	-0.1	1.1	
I	0732	Hypothetical cytosolic protein	hycp	S		1.4	
II	0045–6	Hypothetical protein-Hypothetical protein	hyp/hyp	S			
Posttranslational modification, protein turnover and chaperones							
I	0816	ATP-dependent clp protease ATP-binding subunit	clpA	S	1.0	0.8	1.5
II	0932	Glutaredoxin	nrdH	S		1.1	-0.3
Defense mechanisms							
I	0926	Multidrug resistance locus	emrA			2.2	
General function prediction							
I	1143	Metal-dependent hydrolase (β-lactamase)	mbl	S	-0.1	0.9	
I	1282	Glycine cleavage T protein (aminomethyltransferase)	gcvT	S	1.2	0.4	
I	1487	Colicin V production protein	colV	S	1.2	0.5	0.8
I	1499	Pirin (cupin)	pirA	S	-0.1	1.3	1.7
I	1867	Florfenicol resistance protein	nifB/elp3	S	0.4	0.5	
Carbohydrate transport and metabolism							
I	2031	Phosphocarrier protein HPr	ptsH	S	-0.3	0.9	0.4
II	1095	L-fuculose phosphate aldolase	sbgE	S	0.7		2.2*
Lipid transport and metabolism							
Coenzyme transport and metabolism							
I	0657	Outer membrane receptor (TonB-dependent transport)	btuB	S	0.2	0.6	

^a ^S v. R denotes smooth versus rough colony morphology

^b ^*In vitro *growth: 1:1 mixtures of 16 M and select mutants used to inoculate culture to evaluate growth defects of the mutant. The output ratios are corrected for variance from the input ratios (approximately 1.0) and presented as a log_10 _value, also refrerred to as Δlog_10_. Macrophage survival is presented as a log_10 _of the ratio of recovery 16 M to mutant which may also be presented as Δlog_10_.

^c ^Statistical analysis of macrophage and mouse splenic clearance revealed significant differences among means using 1-way ANOVA, Dunn's multiple comparisons using a non-attenuated mutant revealed statistically valid differences with *p*-values of 0.05 (*), 0.01 (**) and 0.001 (***).

^d ^Mouse splenic clearance: 1:1 mixtures of 16 M and select mutants were used to inoculate mice i.p. to evaluate degree of *in vivo *attenuation of survival at one week.

/ Indicates intergenic insertion between two genes.
